# An artificial intelligence-enabled smartphone app for real-time pressure injury assessment

**DOI:** 10.3389/fmedt.2022.905074

**Published:** 2022-09-23

**Authors:** Chun Hon Lau, Ken Hung-On Yu, Tsz Fung Yip, Luke Yik Fung Luk, Abraham Ka Chung Wai, Tin-Yan Sit, Janet Yuen-Ha Wong, Joshua Wing Kei Ho

**Affiliations:** ^1^ Laboratory of Data Discovery for Health Limited (D24H), Hong Kong Science Park, Hong Kong SAR, China; ^2^ School of Biomedical Sciences, Li Ka Shing Faculty of Medicine, The University of Hong Kong, Pokfulam, Hong Kong SAR, China; ^3^ Department of Emergency Medicine, School of Clinical Medicine, Li Ka Shing Faculty of Medicine, The University of Hong Kong, Pokfulam, Hong Kong SAR, China; ^4^ School of Nursing, Li Ka Shing Faculty of Medicine, The University of Hong Kong, Pokfulam, Hong Kong SAR, China; ^5^ School of Nursing / Health Studies, Hong Kong Metropolitan University, Ho Man Tin, Hong Kong SAR, China

**Keywords:** wound assessment, pressure injury, object detection, mHealth, digital health, bedsore, artificial intelligence, deep learning

## Abstract

The management of chronic wounds in the elderly such as pressure injury (also known as bedsore or pressure ulcer) is increasingly important in an ageing population. Accurate classification of the stage of pressure injury is important for wound care planning. Nonetheless, the expertise required for staging is often not available in a residential care home setting. Artificial-intelligence (AI)-based computer vision techniques have opened up opportunities to harness the inbuilt camera in modern smartphones to support pressure injury staging by nursing home carers. In this paper, we summarise the recent development of smartphone or tablet-based applications for wound assessment. Furthermore, we present a new smartphone application (app) to perform real-time detection and staging classification of pressure injury wounds using a deep learning-based object detection system, YOLOv4. Based on our validation set of 144 photos, our app obtained an overall prediction accuracy of 63.2%. The per-class prediction specificity is generally high (85.1%–100%), but have variable sensitivity: 73.3% (stage 1 vs. others), 37% (stage 2 vs. others), 76.7 (stage 3 vs. others), 70% (stage 4 vs. others), and 55.6% (unstageable vs. others). Using another independent test set, 8 out of 10 images were predicted correctly by the YOLOv4 model. When deployed in a real-life setting with two different ambient brightness levels with three different Android phone models, the prediction accuracy of the 10 test images ranges from 80 to 90%, which highlight the importance of evaluation of mobile health (mHealth) application in a simulated real-life setting. This study details the development and evaluation process and demonstrates the feasibility of applying such a real-time staging app in wound care management.

## Introduction

Caring for pressure injury (PI) in the community, especially for elderlies with limited mobility, has been emerging as a healthcare burden in recent years due to the ageing population (1–3). These injuries are caused by unrelieved pressure occurring over dependent bony prominences, such as the sacrum, the ankle or the heel, as well as shear forces and friction (4). Importantly, PI is preventable and treatable with early diagnosis and proper wound management.

Considering PI conditions and healing progress, National Pressure Injury Advisory Panel (NPIAP) have a defined staging system — stage 1, stage 2, stage 3, stage 4, unstageable, deep tissue pressure injury, and mucosal membrane pressure injury — to guide wound care specialists, clinicians and nurses for treatment options (5). The commonly identified tissue types in PI include epithelialisation, granulation, slough, necrosis, and dry eschar. Accurate and efficient assessment of PI helps healthcare professionals offer appropriate interventions and subsequent progress monitoring.

The prevalence of PI is estimated to be 10%–25.9% (6). Frail elderly, bedridden elderly and elderly with incontinence are particularly vulnerable. Initial PI assessment is normally performed by specialist wound care physicians or nurses in hospitals or clinics, followed by daily or weekly follow-up management by community nurses or other providers. Given its financial implication, patients with PI are usually managed by lay home carers, leading to increased risk of wound infection, delayed healing and hospital admissions (7).

Proper PI staging is critical for treatment plan formulation and progress monitoring. We propose a smartphone-based artificial intelligence (AI) application (app) can provide convenient and effective PI assessment support. We reviewed the state-of-the-art in this field and report our group's effort in the development and evaluation of a real-time PI assessment app.

A number of smartphone or tablet apps for wound assessment, using computer vision algorithms based on mathematical models or AI, have been developed (Table 1). Apps can have one-to-many features built-in, including wound size and depth measurement, localisation and segmentation, tissue classification, manual entry of wound characteristics for documentation and monitoring, and more. Apps are typically made for assessing chronic wounds, such as PI or diabetic foot ulcers.

**Table 1:** Summary of the developed wound assessment smartphone or tablet apps.

Name	Academic publication	Main features	Potential limitations	Platform	Device	Availability
Garcia-Zapirain et al.	Garcia-Zapirain et al., 2018 (8)	Pressure injury (PI) image decomposition and segmentation	Segmentation accuracy and processing time can be improved	Android	Tablet	Not found online or in any app store
Zahia et al.	Zahia et al., 2020 (9)	Automatic PI image segmentation and size and depth measurement based on CNN	Requires Structure Sensor attached to iPad	iOS	Tablet	Not found online or in any app store
FootSnap	Yap et al., 2018 (12) Goyal et al., 2019 (13) Cassidy et al., 2022 (14)	Capture standardised images of plantar surface of diabetic feet (2018); localisation of wound in diabetic foot ulcer images (2019); cloud-based framework for storage (2022)	Lacking any of the other key features (e.g., segmentation / tissue / staging)	Android or iOS	Smartphone or Tablet	Not found online or in any app store
MOWA	Kositzke et al., 2018 (15)	Tissue classification of wound	Method and accuracy unknown; segmentation done manually	Android or iOS	Smartphone or Tablet	Paid
Fraiwan et al.	Fraiwan et al., 2018 (11)	Early detection of diabetic foot ulcers using thermal imaging	Accuracy unknown; external thermal camera required	Android	Smartphone	Not found online or in any app store
SmartWoundCare	Friesen et al., 2013 (16)	Electronic documentation of chronic wounds	No mathematical or machine learning-based features	Android	Smartphone or Tablet	App is freely available
imitoWound	n/a	Wound documentation and measurement	Measurement requires paper-based calibration marker	Android or iOS	Smartphone or Tablet	App is freely available, but sensor for measurement is not
KroniKare	n/a	Capture 3D image of wound; dashboard for wound documentation; wound complication detection; measure wound size; AI-based classification of seven tissue types	Method and accuracy unknown; external device attached to smartphone required	Android or iOS	Smartphone	Availability upon request
CARES4WOUNDS	Chan et al., 2022 (21)	Wound size and depth measurement; AI-based tissue classification; prediction of infection likelihood; output of a wound score; wound documentation and monitoring; wound dressing recommendation based on treatment objectives	Method and accuracy unknown beyond wound size measurement	iOS	Smartphone	Availability upon request
Orciuoli et al.	Orciuoli et al., 2020 (18)	Wound size measurement and AI-based staging classification	A limited set of training images (62 total) and evaluation only reported for the training set	Android	Smartphone	Not found online or in any app store

Some apps require a portable device or component in addition to the smartphone or tablet itself. Garcia-Zapirain et al. (8) implemented a toroidal decomposition-based segmentation algorithm for PI into a tablet app that optimises the treatment plan. The same team later implemented a convolutional neural network (CNN)-based method in a tablet app to automatically segment PI wounds and measure wound size and depth (9). It is important to note that the app requires a Structure Sensor mounted on an iPad to perform the analysis. The imitoWound app (10) was developed for wound documentation and calculation of physical properties of a wound, including wound length, width, surface area, and circumference; these measurements require the use of their custom paper-based calibration markers. Fraiwan et al. (11) proposed an app that makes use of a portable thermal camera (FLIR ONE) for early detection of diabetic foot ulcers based on the Mean Temperature Difference (MTD). This smartphone app only works when attached to the external thermal imaging camera. The accuracy of the method was not benchmarked.

Apps can be conveniently and widely used if they are designed to run on a smartphone or tablet alone. Yap et al. (12) developed an app for assessing diabetic foot ulcers called FootSnap, which the initial version captured standardised images of the plantar surface of diabetic feet and worked on iPad only. Later versions of the app (13) were trained with a CNN to localise wounds in diabetic foot ulcer images, deployed on Android platform, and proposed using a cloud-based framework to store the images taken from the app (14). Kositzke and Dujovny (15) used a wound analyser software called MOWA to perform tissue classification in Android or iOS platforms and smartphone or tablet devices. However, the classification method and accuracy are both unknown, and the wound segmentation must be done manually. Friesen et al. (16) developed an Android smartphone or tablet app titled SmartWoundCare, which was used for electronic documentation of chronic wound management, especially for pressure ulcers. Instead of AI, the app focuses on functions such as telemedicine, wound record management, and tutorials for non-specialists. The group also proposed methods for detecting wound size and colour (17), but as far as publicly available, these are not currently in the SmartWoundCare app. Orciuoli et al. prototyped a smartphone app for size measurement and staging classification based on deep learning. Their staging classifier was trained on a limited set of images (62 total), and the evaluation was only done on the training set (18).

More ambitious apps have many of the mentioned features, whether or not an external device is required. KroniKare (19) provides an AI-based assessment of the wound. A custom-made scanning device, in addition to the smartphone, is required to take a 3D image of the wound. It measures the wound size, identifies tissue types, provides an integrated dashboard for documentation, and detects wound complication such as undermining or infection. Yet, peer review evaluation of its performance is lacking. CARES4WOUNDS (20) also provides AI-based wound assessment in tissue classification, automated wound size and depth measurement, prediction of infection likelihood, and an output of a wound score. The app accepts manual entry of wound characteristics related to documentation and monitoring and recommends wound dressing treatment plans from user-decided treatment objectives using a decision tree. The app works only on later versions of the iPhone (11+) with an integrated depth scanner. A clinical study found the wound size measurement of CARES4WOUNDS very accurate for diabetic foot ulcers (21). Other features of the app about classification or various prediction or score-based outputs have not yet been reported in the literature.

Out of the smartphone or tablet-based wound assessment apps we reviewed here, only Orciuoli et al. directly perform PI staging classification. Other apps mostly focus on size measurement or tissue segmentation, probably due to these features being more intuitively identifiable from the wound image. Staging a PI requires a trained professional, and staging results are not completely free from bias. To build on the existing study, we sought to create our own PI staging classification smartphone app using a larger set of images, further increased by data augmentation, and to more objectively evaluate the classifier using validation and testing sets (both were not included in the training). The challenges of building and evaluating a smartphone PI staging classifier include the requirement of an expert wound specialist to generate the ground truth, the different lighting, angle, wound relative size, and overall quality of the images, and further differences of these factors after a wound image is captured by a smartphone camera. In this article, we describe the process of developing an Android smartphone app that can automatically detect and classify PI. We also present a systematic evaluation of the performance of this app.

## Methods

The main objective of this study is to develop and evaluate a practical, real-time PI assessment smartphone app. As a pilot study, we developed an AI-based app that was trained with publicly available PI images. The data set was based on a publicly available GitHub repository (22), consisting of images from the Medetec Wound Database (23) and other public sources. After manual inspection to remove images that do not contain PI, the data set has 190 images from pressure ulcer images, including stage 1 (38 photos), stage 2 (35 photos), stage 3 (38 photos), stage 4 (42 photos) and unstageable (38 photos). We manually identified the wound using boundary boxes and annotated each wound based on the definition of the staging system by NPIAP (5). The annotation was reviewed and confirmed by an experienced (>10 years) wound nurse, co-author Tin-Yan Sit. She is a registered nurse in Hong Kong, and an enterostomal therapist who is a member of the WCET (World Council of Enterostomal Therapists). Members of WCET are nurses who provide specialised nursing care for people with ostomy, wound and continence needs; she is also a member of Hong Kong Enterostomal Therapist Association.

Data augmentation was performed by Roboflow (24). Each image in the data set was subjected to three separate transformations—flipping, cropping, and brightening—to create two more images. We then randomly split the data into training and validation sets such that no image in the training set shares the same source as any image in the validation set. To further enhance the size of the training set, we performed another round of data augmentation on the training set with three separate transformations: rotation, shear, and exposure. In total, the training set contains 1,278 images, and the validation set contains 144 images.

We used a high-performance open source object detection and classification system, YOLOv4 (25), as the core AI component in our app. You Only Look Once (YOLO) is an object detection system for real-time processing (26) and uses a neural network to predict the target object's position and class score in a single iteration. Carrión et al. (27) used 256 images of the wound-inflicted lab mice in the YOLO model for object detection. Han et al. (28) proposed a real-time detection and classification software for Wagner grading of diabetic foot wounds. In addition, they found that YOLO achieved a better speed and precision trade-off than other wound localisation models like Single Shot MultiBox Detector (SSD) and Faster R-CNN. These studies show that YOLO is a suitable model for detecting or locating a wound.

Here, we designed a real-time wound detection and classification program based on YOLO's deep-learning training capability and implemented it in a smartphone app (29). We trained YOLO version 4 (v4) (25) on open-source PI images and performed the detection and classification. Following the implementation step in YOLOv4, we first configured cuDNN, a deep learning Graphical Processing Unit (GPU) acceleration library based on CUDA. Then, we installed an open-source neural network framework called Darknet, which was written in C and CUDA technology. Computations of YOLOv4 are performed on GPU. All the annotated wound images in the training set were exported in the YOLO Darknet format. We trained the YOLOv4 detector by using the default training parameter setting. After training the model, we saved the weights file every 1,000 iterations, for a total of 10,000 iterations, the best weights, and the last weights.

**Figure 1:**
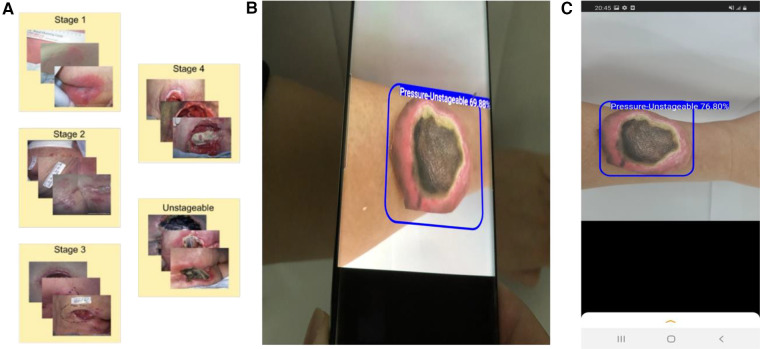
Development of a smartphone app for wound assessment. (**A**) Collection and annotation of images of pressure injury wounds. (**B**) Application of our smartphone app to detect and classify PI. (**C**) A screenshot showing how a printed wound image is automatically detected and classified using the app.

The trained YOLOv4 model was then converted to a TensorFlow Lite (TFLite) model for deployment in an Android device (30). We further built a graphical user interface to make this app user-friendly (Figure 1).

The accuracy of our model is calculated by:

$$\frac{TP}{TP+FN+FP+FN}=\frac{TP}{\mathrm{total}}$$

The Matthews Correlation coefficient (MCC) for individual classes (A vs. not A) is calculated by:

$$\begin{array}{rl} & MCC_{A}=\frac{TP_{A}\times TN_{A}-FP_{A}\times FN_{A}}{\sqrt{\left(TP_{A}+FP_{A}\right)\left(TP_{A}+FN_{A}\right)\left(TN_{A}+FP_{A}\right)\left(TN_{A}+FN_{A}\right)}} \end{array}$$

where A can be stage 1,2,3,4 or unstageable (U).

Bootstrap mean:

Suppose we have an original data set of $x=(x_{1},\ldots ,x_{n})$ with ascending order, we calculated the mean and SD of this original data set.

To create a bootstrap sample, we randomly select the data from the original data set n times and obtain a data set of $x^{\prime }=(x_{1}^{\prime },\ldots ,x_{n}^{\prime })$. This process is repeated 1,000 times. For each bootstrap sample, we calculated the mean. Finally, we obtained the SD of the mean of these 1,000 bootstrap samples.

The 95% confidence interval is calculated as:

$$\left[2\bar{x}-x_{1-\alpha }^{*}\;,\;2\bar{x}-x_{\alpha }^{*}\right]$$

with $\bar{x}$ is the mean of the original data set and $x^{*}$ is the bootstrap means.

In 95% confidence interval,

$$\alpha =\frac{\left(1-95\%\;\right)}{2}$$

We estimate the prediction accuracy of our model based on ten-fold cross-validation (10 fold CV) on the training data set; in addition, we evaluate our model based on a held-out validation set and an independent test set.

## Results

**Figure 2:**
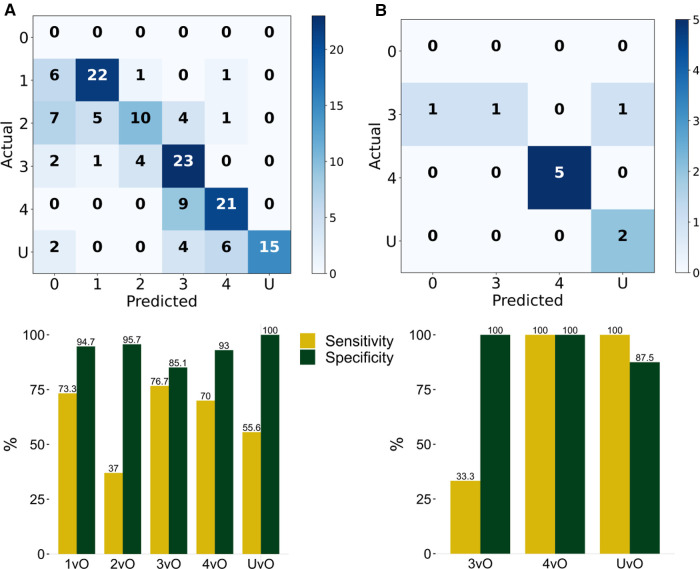
Evaluation of the classification results of the PI stages. Stage 1 (1), stage 2 (2), stage 3 (3), stage 4 (4), and unstageable (**U**) of the trained YOLOv4 model by computing the confusion matrix and corresponding Sensitivity (TP/TP + FN) and Specificity (TN/TN + FP) of (**A**) validation set of 144 photos, and (**B**) testing set of 10 photos. 1vO: Stage 1 vs. Others; 2vO: Stage 2 vs. Others; 3vO: Stage 3 vs. Others; 4vO: Stage 4 vs. Others; UvO: Unstageable vs. Others.

**Figure 3:**
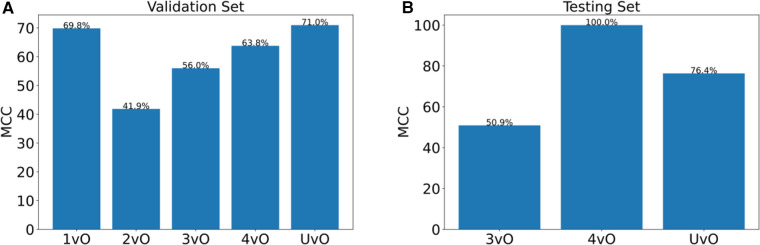
Matthews correlation coefficient (MCC) of (**A**) validation set of 144 photos, and (**B**) testing set of 10 photos. 1vO: Stage 1 vs. Others; 2vO: Stage 2 vs. Others; 3vO: Stage 3 vs. Others; 4vO: Stage 4 vs. Others; UvO: Unstageable vs. Others.

To validate the PI staging classification, we applied our trained YOLOv4 model to the 144 validation PI images. The confusion matrix is shown in Figure 2A. The overall prediction accuracy is 63.2%. We calculated the per-class prediction sensitivity and specificity by comparing the results of each stage against the other stages in the confusion matrix. The per-class prediction sensitivity is variable, at 73.3% (stage 1 vs. others), 37% (stage 2 vs. others), 76.7 (stage 3 vs. others), 70% (stage 4 vs. others), and 55.6% (unstageable vs. others). The per-class prediction specificity is uniformly high, at 94.7% (stage 1 vs. others), 95.7% (stage 2 vs. others), 85.1 (stage 3 vs. others), 93% (stage 4 vs. others), and 100% (unstageable vs. others). We also calculated the per-class prediction Matthews correlation coefficient (MCC) of the model on the same validation set, and the mean MCC across different classes is 60.5%, which is consistent with the accuracy estimate (Figure 3A).

To further test the predictive performance of the model, we collected an additional 10 test images using our local patients. Eight out of 10 images had correct classification (accuracy = 80%). The per-class sensitivity is within 33.3%–100%, and the specificity is within 87.5%–100% (Figure 2B), and the MCC is within 50.9%–100% (Figure 3B).

For estimating the reliability of the metrics of our model, we computed 10-fold cross-validation on the training set images, and for each fold, we computed the prediction accuracy and per-class MCC, as well as the mean and 95% confidence interval across the 10 folds for each metric (Table 2). Our mean 10-fold CV accuracy is 73.3% and MCC are 61.56%, 46.72%, 81.11%, 86.51%, 86.67% in the 1vO, 2vO, 3vO, 4vO, and UvO comparisons, respectively. The MCC estimated from the validation set is slightly lower than the estimates from the 10-fold CV experiment (Table 2). This may be caused by an underestimate of the confidence intervals due to large variance in the MCC estimates of the 10 folds. Nonetheless, the overall accuracy and the trend of the per-class MCC estimates between the 10-fold CV and validation set are quite consistent.

**Table 2:** Accuracy and Matthews correlation coefficient (MCC) of the training set of 1,278 pressure ulcer images by 10-fold cross-validation.

Fold	Accuracy (%)	MCC (%)				
		1vO	2vO	3vO	4vO	UvO
1	51.6	45.6	70	83.7	24.7	45.7
2	69.0	56.9	23.6	83.2	91.3	95.3
3	76.2	51.0	37.4	83.1	100	100
4	81.0	61.4	86.8	90.5	92.9	95.3
5	81.0	85.2	46.4	87.7	97.6	90.6
6	69.0	76.4	20.7	36.9	86.9	93.4
7	74.6	64.2	33.1	80.5	93.4	85.6
8	71.4	51.4	58.4	91.3	88.0	72.5
9	75.5	52.8	27.1	93.5	98.1	93.0
10	83.7	70.7	63.7	80.7	92.2	95.3
Average	73.3	61.56	46.72	81.11	86.51	86.67
SD (bootstrap)	2.734	3.682	6.744	4.845	6.754	4.895
95% confidence interval	[68.39,79.41]	[53.71,68.58]	[33.53,59.56]	[74.02,91.84]	[77.55,100]	[78.96,97.60]

To construct an empirical baseline for comparison, we re-trained the model using the training set images with shuffled class labels (label shuffling done on the level of the original images) and calculated the evaluation metrics on the validation set ten times (Table 3). We recorded a mean shuffling test accuracy of 19.32% and multi-class MCC of −7.06–21.01, which are much poorer than our standard and 10-fold CV results. This result indicates that our trained YOLO model performed much better than a random baseline model, and has indeed learned.

**Table 3:** Accuracy and Matthews correlation coefficient (MCC) of validation set of random label shuffling of training set of 1,278 pressure ulcer images, repeated ten times.

Permutation	Accuracy (%)	MCC (%)				
		1vO	2vO	3vO	4vO	UvO
1	17.4	−6.2	−1.1	1.7	17.4	21.8
2	23.6	−18.1	11.7	11.0	35.3	37.3
3	29.9	−18.8	27.0	35.3	30.5	20.0
4	16.0	−8.7	31.7	−8.7	17.8	22.5
5	18.1	5.5	9.6	−19.4	9.2	19.2
6	17.4	−10.7	−2.6	2.4	40.9	22.5
7	11.8	−18.8	22.8	−10.2	4.6	−17.9
8	20.8	9.3	7.7	4.9	35.3	−4.0
9	16.7	−16.2	14.0	23.1	11.0	14.3
10	21.5	12.1	−7.4	17.6	8.1	25.5
Average	19.32	−7.06	11.34	5.77	21.01	16.12
SD (bootstrap)	1.470	3.636	3.719	4.987	3.874	4.619
95% confidence interval	[16.26,21.94]	[−14.18,0.05]	[4.06,18.71]	[−4.65,14.64]	[13.17,28.52]	[8.37,25.51]

**Figure 4:**
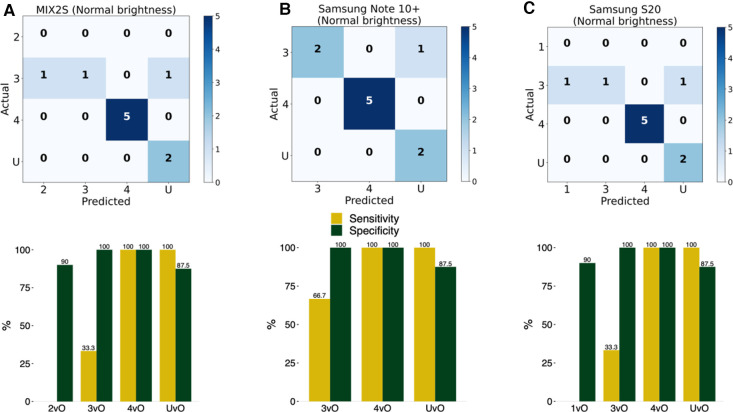
Evaluation of accuracy of the implemented model in an Android smartphone app. The detection and classification of the printed wound images in the 10 photos in the test set using the smartphone app with three different Android phones at normal (125 lux) brightness level: (**A**) MIX2S, (**B**) Samsung Note 10+, and (**C**) Samsung S20. 1vO: Stage 1 vs. Others; 2vO: Stage 2 vs. Others; 3vO: Stage 3 vs. Others; 4vO: Stage 4 vs. Others; UvO: Unstageable vs. Others.

To test the practical utility of the smartphone app, as opposed to just the performance of the ML model, we printed the 10 test PI images on blank white papers and adhered the cut-out of the wound image on the skin of a forearm. The idea is that this design provides a more realistic evaluation strategy for practical performance of the app. This design allows us to test the impact of the ambient environment, such as brightness, as well as the stability across different phones which have distinct cameras. In this test, we placed the smartphone app at a distance to enable the wound to be clearly seen inside the screen. We tested three Android smartphone models (Figures 4A–C) across two brightness levels of indoor illumination levels (Table 4). In particular, we noted that the classification accuracy varies between 80% to 90% depending on phone model and ambient brightness (Figure 4). The estimated sensitivity and specificity are also shown in Figure 4 and Table 4. These findings indicated that the app was reasonably robust in realistic situations.

**Table 4:** Per-class specficity and sensitivity of the implemented model in three different android phones at two different ambient brightness levels on the test set images.

	Sensitivity (%)	Specificity (%)
Phone model (ambient brightness level)	1vO	2vO	3vO	4vO	UvO	1vO	2vO	3vO	4vO	UvO
MIX2S (normal)	–	–	33.3	100	100	–	90	100	100	87.5
MIX2S (dim)	–	–	33.3	100	100	–	90	100	100	87.5
Samsung Note 10+ (normal)	–	–	66.7	100	100	–	–	100	100	87.5
Samsung Note 10+ (dim)	–	–	33.3	100	100	–	90	100	100	87.5
Samsung S20 (normal)	–	–	33.3	100	100	–	90	100	100	87.5
Samsung S20 (dim)	–	–	33.3	100	100	–	90	100	100	87.5

Normal is brightnes at 125 lux and dim is brightness at 58 lux.

## Discussion

This study reports the development and evaluation of the first AI-enabled smartphone app for real-time pressure injury staging assessment. Other app developers have considered size and depth measurement, wound segmentation, tissue classification, treatment recommendation, and the applicable wound type (PI, diabetic foot ulcer, or others). We also plan to explore some of these features in the future. Still, our current proposed AI-based app, which is built upon open-source PI images verified by wound care nurses, provides a reasonable PI staging support tool for lay carers. With early diagnosis and proper management of PI, wound infection and hospital admission are prevented.

We identified several issues that can be improved. First, the realistic conditions testing of the app was only done on printed images, while testing on real patient wounds will be needed to ensure robustness of the results. Further technical development, such as utilising the flash light in a smartphone, may further improve the sensitivity and specificity of real-time image capture. Second, high standard performance including accuracy, sensitivity and specificity, and incorporation of features such as speed, operating efficiency, and convenience in retrieving patient medical records, as well as comparing and contrasting PI parameters for healing monitoring, are necessary for more professional use. Third, our app is used on Android platform only, and should therefore aim to be compatible with both Android and iOS devices. Fourth, with the real-time function, the app can be further optimised to inter-operate with existing tele-medicine platforms for supporting remote medical consultations.

In the community application, AI wound assessment in smartphones has the potential to perform early wound diagnosis, optimise wound management plans, reduce healthcare costs, and improve patients' quality of life in residential homes by the carers. Our newly developed app can perform real-time PI assessment without the need to use additional hardware (e.g., thermal camera and structure sensor) and internet access, making it easily deployable in the community. Nevertheless, the practicality of wound assessment assisted by an AI app can depend on the level of acceptance of such technology from health workers.

As a feasibility study, we mostly made use of publicly available data for training and validation of the machine learning system. We collected a small set of additional test photos for independent validation. Still, the number of images is still relatively small. In the future, we will further collect high quality PI images for training and evaluation. In addition, we should conduct an evaluation study on real patients and a usability of nurses and wound specialists.

In medical and nursing education or specialist training, these apps have potential to serve as an educational tool for learners to practice. As the apps can detect both photos or real wound, it enhances teachers' resources for teaching wound assessment and management. Also, technology-based pedagogy may enhance students' learning motivation and arouse their interest to practice, which will maximize their learning performance.

We demonstrated that the detection of a pressure ulcer by the object detection model YOLO. The app stages pressure ulcers with our limited training and test sets. The results have indicated that we can have a correct classification of the staging level by YOLO if we have enough training set of the pressure ulcer images. We also implemented our trained YOLO into an Android app. The app can detect the wound successfully in real-time by the phone camera. The size, dimensionality, and distance from the phone camera are the factors that affected the classification results. Overall, in this work, we developed a real-time smartphone or tablet app to detect and classify the staging of pressure injuries. It provides the foundation of pressure injuries assessment with an object detection app. Furthermore, our proposed technology for wound assessment may help to prevent and recover pressure injuries without leading to wound infection and hospital admission.

## Data Availability

Publicly available datasets were analyzed in this study. This data can be found here: https://github.com/mlaradji/deep-learning-for-wound-care/tree/master/data/google-images-medetec-combined/pressure-ulcer-images.
